# Disease-Specific Associations of Physical Activity in Patients with Rheumatoid Arthritis and Systemic Lupus Erythematosus

**DOI:** 10.1159/nim/adxag003

**Published:** 2026-09-05

**Authors:** Justus Mörstedt, Lev Lipovsky, Benjamin Graul, Olga Seifert

**Affiliations:** Department of Rheumatology, University Hospital Leipzig, Leipzig, Germany

**Keywords:** Physical activity, Rheumatoid arthritis, Systemic lupus erythematosus, Fibromyalgia, Patient-reported outcomes

## Abstract

**Introduction:**

Patients with inflammatory rheumatic diseases exhibit reduced physical activity (PA), contributing to increased cardiovascular risk and functional decline. While PA is recommended in both rheumatoid arthritis (RA) and systemic lupus erythematosus (SLE), variables associated with PA remain poorly understood. Therefore, this study explored shared and disease-specific associations with PA in RA and SLE.

**Methods:**

In this cross-sectional study, patients with RA (*n* = 200) and 94 SLE (*n* = 94), fulfilling ACR classification criteria, were recruited from the rheumatological outpatient clinic of the University Hospital Leipzig. Sociodemographic data were obtained from electronic records. Patient-reported outcome measures assessed comorbidities, PA, and health-related quality of life. PA was quantified using the International Physical Activity Questionnaire-Long Form. Associations were examined using multivariable logistic regression models.

**Results:**

Patients with RA showed lower physical performance (*p* < 0.001) but higher PA levels than SLE (*p* < 0.001). In RA, fibromyalgia remained the only statistically significant variable and was associated with lower PA (OR = 0.001, 95% confidence interval [CI]: < 0.001–0.289; *p* = 0.017). In SLE, no independent predictors were identified; fibromyalgia (OR = 0.374, *p* = 0.209) and depressive symptoms (OR = 0.341, *p* = 0.063) showed nonsignificant trends. Self-reported PA and objective walking performance were moderately associated in both groups (RA: *R*^2^ = 0.19; SLE: *R*^2^ = 0.12).

**Conclusion:**

Associations with PA differ between RA and SLE. In RA, fibromyalgia is associated with lower PA, whereas in SLE, PA reflects a multifactorial interplay of functional and psychological factors, supporting disease-specific approaches to PA promotion.

## Introduction

Rheumatoid arthritis (RA) and systemic lupus erythematosus (SLE) are chronic systemic autoimmune inflammatory conditions, associated with reduced quality of life as well as psychological and musculoskeletal impairments [1]. In comparison to the general population, patients with RA and SLE have an increased risk of cardiovascular diseases (CVDs) and suffer from a variety of comorbidities [2]. CVD account for a substantial proportion of the increased mortality in rheumatic diseases (RDs) and are considered the leading cause of death [3, 4]. Consequently, lifestyle modifications, particularly a healthy diet and regular physical activity (PA), have become central elements in the prevention and management of CVD and comorbidities [2, 5]. Chronic inflammation, pain, depression, and functional limitations associated with RDs often restrict patients in their ability to engage in PA [6]. Although the pathogenesis of mood disorders is not yet fully understood, growing evidence indicates an immune-mediated component, characterized by elevated cytokines such as IL-6 and TNF-α that interact with stress-responsive endocrine pathways [7–9]. Evidence supports that individuals with a pronounced inflammatory profile show reduced responsiveness to conventional antidepressant therapies [7]. As depression can increase pain perception and pain may exacerbate depression, this neuropsychiatric interplay may further contribute to physical inactivity in RDs [6, 10]. Patients with RDs fail to reach recommended daily step counts, neither those proposed for the general population nor the lower thresholds suggested for people with chronic conditions [1]. This indicates that RDs are commonly associated with reduced PA. Given the independent cardiovascular risk linked to inflammatory conditions in RDs, the European Alliance of Associations for Rheumatology (EULAR) states that patients should be reassured that exercise is safe, beneficial, and effective at any stage of the disease, regardless of prior activity levels [5]. Moreover, the World Health Organization (WHO) recommends that guidelines for a healthy lifestyle, although developed for the general population and targeting 150 min/week of moderate-to-vigorous PA, can also be applied [5].

Lifestyle interventions are generally considered beneficial, but their true effectiveness in patients with RDs remains difficult to determine, as existing studies often face methodological limitations and heterogeneous populations, making it challenging to account for factors such as disease severity, comorbidities, and functional capacity that may strongly influence outcomes. Additionally, most research on exercise and PA in RDs has focused on RA, whereas evidence in SLE remains less consistent [5]. This imbalance partly reflects the greater heterogeneity and fluctuating clinical course of SLE, which complicates the evaluation of PA effects and the formulation of standardized recommendations. In this context, patient-reported outcome measures (PROMs) play an essential role, as they capture patients’ perceptions of health outcomes and disease impact [11]. Especially in RDs, PROMs serve as a complementary measurement in clinical trials, when used as validated and reliable tools to appropriately assess disease status and comorbidities [11–13].

Further patient-centered research is needed to clarify how disease-related factors are associated with PA behavior and functional performance. While evidence on PA in RA is relatively consistent, findings in SLE remain limited and partly contradictory. It is still unclear how self-reported patient characteristics relate to subjectively assessed PA and objectively measured physical performance, and whether these associations differ when examined using multivariable models. The associations of comorbidities with self-reported PA and the relationship between subjective PA assessments and objectively measured physical performance have not been clearly established. To address these gaps, the present study compares RA and SLE to identify shared and disease specific associations with physical (in)activity and functional performance.

## Materials and Methods

### Participants and Data Collection

Two hundred patients with RA and 94 with SLE, diagnosed according to the established American College of Rheumatology (ACR) classification criteria applicable at the time of diagnosis, were recruited from the rheumatological outpatient clinic of the University Hospital Leipzig. Both groups were assessed regarding sociodemographic, clinical, functional, and psychological variables and analyzed separately to identify disease-specific patterns and correlates of PA. Baseline characteristics are presented in Table 1. Both studies were approved by the Local Ethics Committee (approval: RA 363/18-ek; SLE 035/16-ek).

**Table 1.** Baseline characteristics of patients with RA and SLE

Variable	RA (*n* = 200)	SLE (*n* = 94)	*p* value
Age, years	66.2±11.4	52.9±15.8	<0.001
Sex (female), *n* (%)	145 (72.5)	87 (92.6)	<0.001
Disease duration, years	18.2±12.6	21.2±9.6	0.043
HAQ score	1.0±0.8	0.5±0.6	<0.001
2-min walking distance	126.9±37.8	164.6±34.2	<0.001
BDI-II	8.8±7.3	10.7±7.9	0.038
PainDetect	10.4±7.0	11.3±7.3	0.410
FACIT-Fatigue	–	34.5±10.2	–

Values are presented as mean ± SD or *n* (%). *p* values refer to between-group comparisons.

RA, rheumatoid arthritis; SLE, systemic lupus erythematosus; HAQ, Health Assessment Questionnaire; BDI-II, Beck Depression Inventory-II; FACIT-Fatigue, Functional Assessment of Chronic Illness Therapy-Fatigue Scale.

### Clinical Assessment

Sociodemographic factors (age, sex, and smoking status) were retrieved from the electronic patient database. Information on current medication, including glucocorticoid therapy, was obtained from the medical records. Low-dose glucocorticoid therapy was defined as prednisone <7.5 mg/day. The following indices were utilized: Beck Depression Inventory Second Edition (BDI-II) for the assessment of depressive symptoms [14], Disease Activity Score in 28 joints (DAS28-CRP) for RA [15, 16], and the Systemic Lupus Erythematosus Disease Activity Index (SLEDAI-2K) [17] for SLE to measure disease activity. Due to sparse data in higher activity categories, disease activity was dichotomized into low and moderate-to-high disease activity (RA: DAS28-CRP >3.2; SLE: SLEDAI-2K ≥6) [18, 19]. Functional impairment was assessed using the Health Assessment Questionnaire-Disability Index (HAQ-DI) [20], neuropathic pain components using the PainDETECT Questionnaire (PD-Q) [21], and health-related quality of life using the Short Form-36 Health Survey (SF-36) [22]. Disease duration was calculated as the date of initial diagnosis and the date of data extraction from the hospital’s electronic patient database. Fibromyalgia syndrome was defined according to the ACR 1990 classification criteria [23]. Objective physical performance was evaluated using the 2-minute walk test [24]. The Functional Assessment of Chronic Illness Therapy-Fatigue Scale (FACIT-Fatigue) was performed only in the SLE cohort as part of the disease-specific assessment [25] and was therefore not analyzed for RA patients.

### Assessment of Physical Activity

PA was assessed using the standardized International Physical Activity Questionnaire-Long Form (IPAQ-LF), capturing self-reported PA during the previous 7 days across 4 domains (work, transportation, domestic activities, and leisure time). Activities were categorized by intensity (walking, moderate, vigorous) and expressed as metabolic equivalent minutes per week (MET-min/week) [26].

PA was analyzed using two IPAQ classifications: a standard dichotomous grouping (low vs. moderate/high) and an alternative grouping contrasting low and moderate with high (health enhancing) PA. The alternative grouping was applied to improve model stability and to distinguish patients achieving health-enhancing PA levels from those remaining below [27]. Both IPAQ dichotomization approaches were applied consistently in RA and SLE to ensure methodological comparability between cohorts.

### Statistical Analysis

Descriptive analyses were performed separately for RA and SLE. Continuous variables are presented as means and standard deviations and categorical variables as frequencies or percentages. Between-group differences were assessed to characterize the study population and identify diagnosis-specific patterns. Normal distribution was evaluated using the Kolmogorov-Smirnov and Shapiro-Wilk tests. Depending on data distribution, group comparisons were conducted using independent-samples *t* tests or Mann-Whitney U-tests.

Associations between PA (IPAQ classification) and potential predictors were examined using binary logistic regression. IPAQ results were dichotomized, and odds ratios (ORs) with 95% confidence intervals (CIs) were reported. Variables with *p* ≤ 0.10 in univariate analyses were considered for multivariable modeling. Given the cross-sectional design, ORs were interpreted as prevalence ORs.

Diagnosis-specific multivariable logistic regression models were constructed based on univariate results and clinical relevance with a focus on assessing associations. Models were built stepwise to assess potential confounding across functional, psychological, and clinical domains. Model fit was evaluated using the omnibus test of model coefficients, Nagelkerke *R*^*2*^, the Hosmer-Lemeshow goodness-of-fit test, and classification accuracy. To assess model stability, simplified models including key functional and clinical predictors were additionally fitted. Analyses were conducted using both the standard IPAQ grouping (low vs. moderate and high) and an alternative grouping (low and moderate vs. high). Multivariable analyses were conducted using complete cases, as not all patients completed all questionnaires and functional assessments. To assess the potential impact of sparse data bias and quasi-complete separation, additional sensitivity analyses using Firth penalized logistic regression were performed for the final RA model. Statistical analyses were conducted using IBM SPSS Statistics (IBM Corporation, Armonk, NY, USA).

## Results

Patients with RA and SLE differed in baseline demographic and clinical characteristics, functional performance (Fig. 1), and physical activity. Patients with RA were older than those with SLE and included a higher proportion of men (both *p* < 0.001, Table 1). Disease duration was longer in SLE (*p* = 0.043), whereas functional limitation was more pronounced in RA (*p* < 0.001). Patients with RA also showed reduced functional exercise capacity in the 2-minute walk test (*p* < 0.001). Depressive symptom scores were higher in SLE (*p* = 0.038). No between-group difference was observed for PainDetect scores. Individual SF-36 subscale results are presented in online supplementary Table S1.

**Fig. 1.**
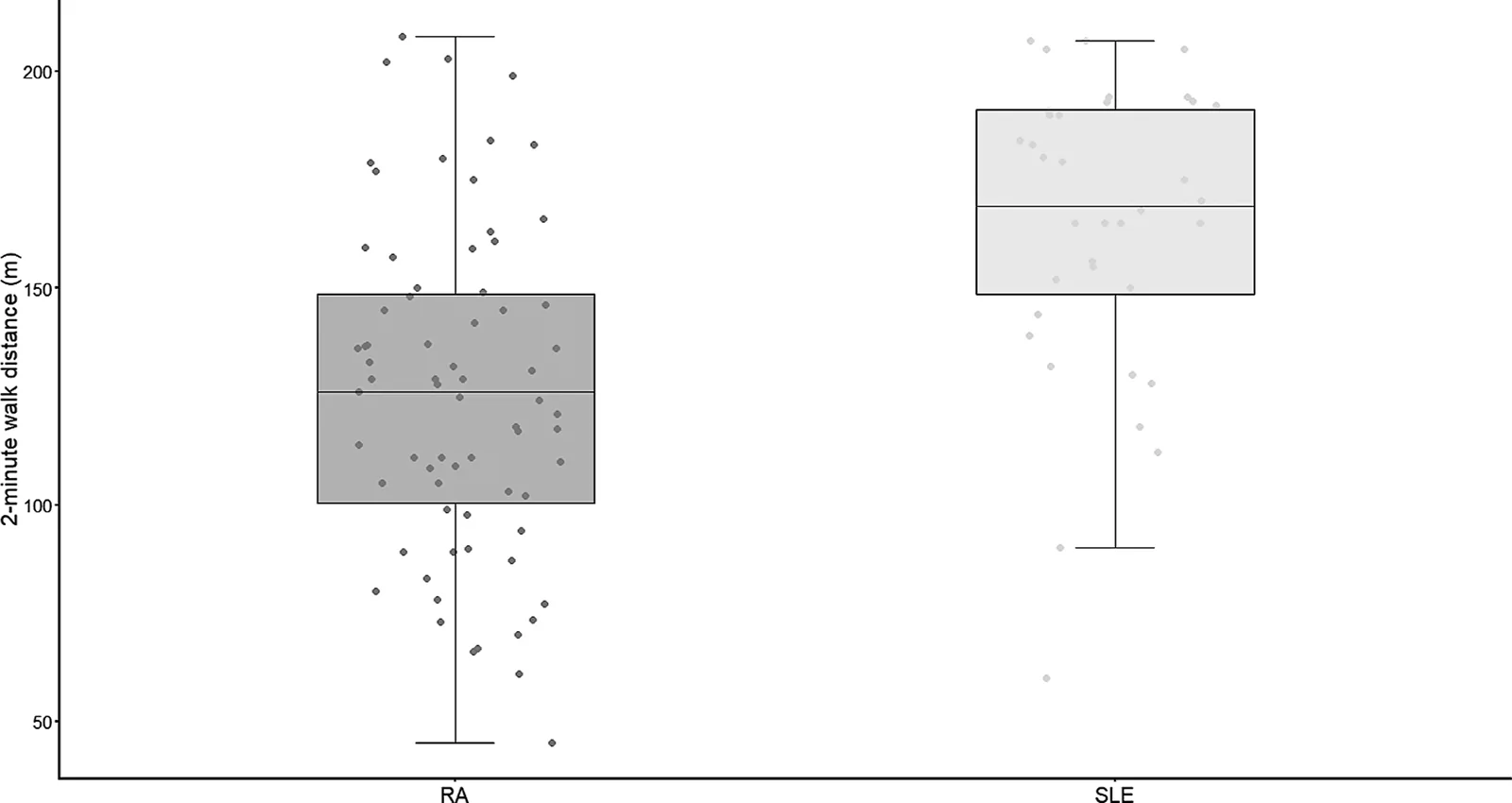
Distribution of 2-minute walk test performance in patients with rheumatoid arthritis and systemic lupus erythematosus. Box plots display the median, interquartile range, and range; individual dots represent single patient values. RA, rheumatoid arthritis; SLE, systemic lupus erythematosus; 2MWT, 2-minute walk test.

In both cohorts, DMARD-based therapy constituted the mainstay of treatment. Patients with SLE were predominantly treated with DMARD monotherapy, most commonly hydroxychloroquine. Glucocorticoid use was frequent, largely at low doses. Biological therapies were rarely prescribed. RA patients more often received conventional synthetic DMARDs in combination regimens, and targeted synthetic or biologic DMARDs were commonly used, typically alongside conventional synthetic DMARDs. Glucocorticoid use was more prevalent in SLE than in RA, whereas targeted synthetic or biologic DMARD therapies were more frequent in RA.

Objective functional performance differed between groups. A higher proportion of patients with RA performed below the age-adjusted normative reference value [28] in the 2-minute walk test compared with patients with SLE (χ^2^ = 11.98, *p* < 0.001, shown in Fig. 2). Patients with RA reported higher levels of PA compared to those with SLE (both IPAQ classifications *p* < 0.001, Table 2). Functional impairment, as assessed by HAQ categories, was more pronounced in RA patients (*p* < 0.001), while dichotomized disease activity did not differ between groups (*p* = 0.919, Table 2). Detailed disease activity categories are provided in online supplementary Table 2.

**Fig. 2.**
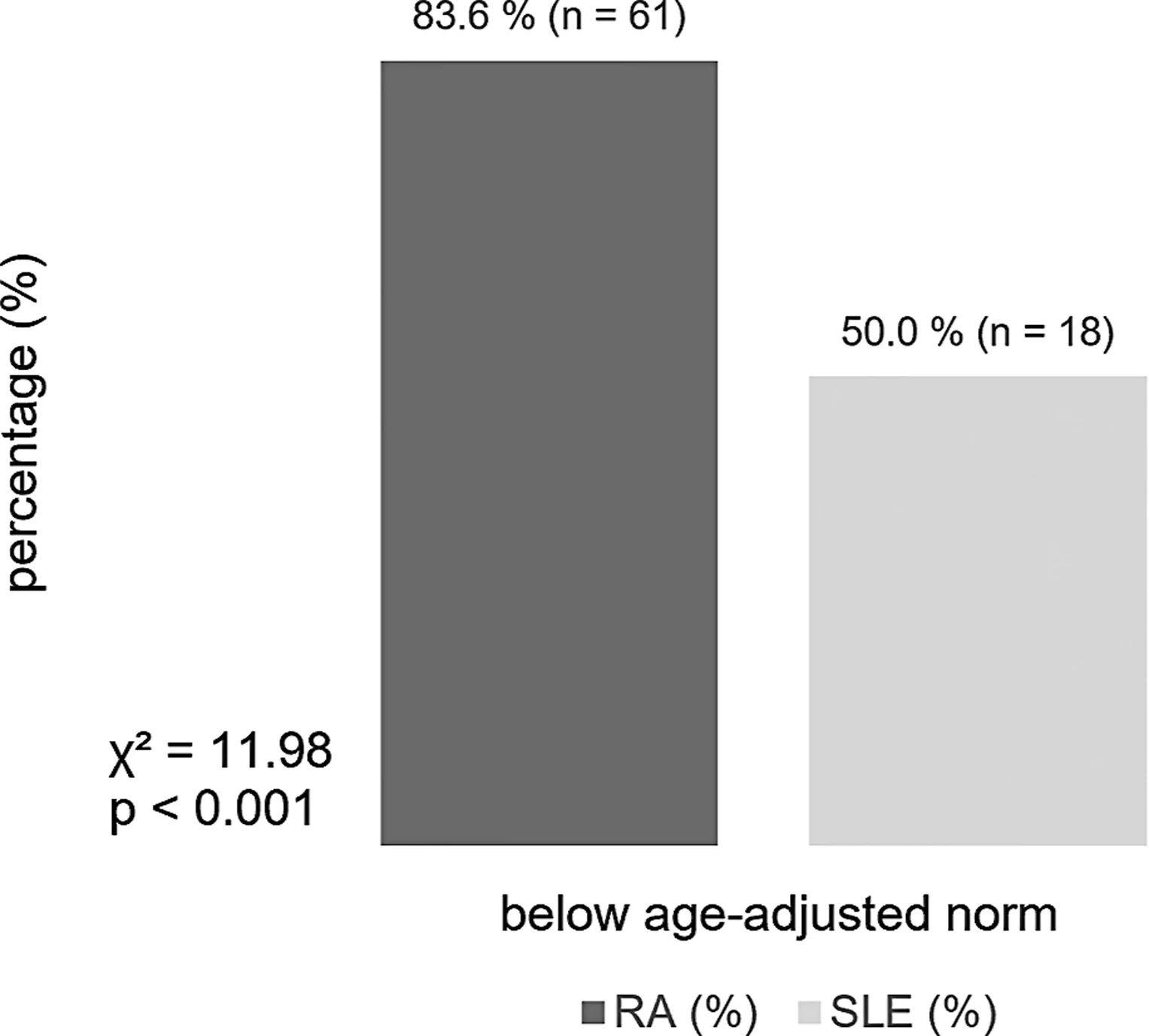
Proportion of patients within and below the age-adjusted normative reference value in the 2-minute walk test. Group differences between patients with RA and SLE were analyzed using the χ^2^ test. RA, rheumatoid arthritis; SLE, systemic lupus erythematosus.

**Table 2.** Distribution of physical activity and functional parameters in RA and SLE

Variable	Category	RA, %	SLE, %	χ^2^	*p* value
IPAQ activity level	Low	14.4	41.3	23.773	<0.001
​	Moderate	34.8	28.8	​	​
​	High	50.8	30.0	​	​
IPAQ dichotomized	Inactive	14.4	41.3	22.921	<0.001
​	Active	85.6	58.8	​	​
IPAQ alternative	Lower PA	49.2	70.0	9.748	0.002
​	Higher PA	50.8	30.0	​	​
HAQ	Mild	56.1	84.0	21.613	<0.001
​	Moderate	31.7	12.8	​	​
​	Severe	12.2	3.2	​	​
Disease activity	Low	60.9	61.5	0.010	0.919
​	High	39.1	38.5	​	​

Values are given as percentages. *p* values are based on χ^2^ tests comparing both groups.

RA, rheumatoid arthritis; SLE, systemic lupus erythematosus; IPAQ, International Physical Activity Questionnaire; PA, physical activity; HAQ, Health Assessment Questionnaire.

Self-reported PA was positively associated with walking performance in both RA (slope = 0.0050, *R*^2^ = 0.19) and SLE (slope = 0.0059, *R*^2^ = 0.12). Across the range of reported activity levels, overall walking performance remained higher in SLE (shown in Fig. 3). Psychological characteristics were largely comparable between groups. Fibromyalgia was more frequent in SLE (*p* = 0.031, Table 3).

**Fig. 3.**
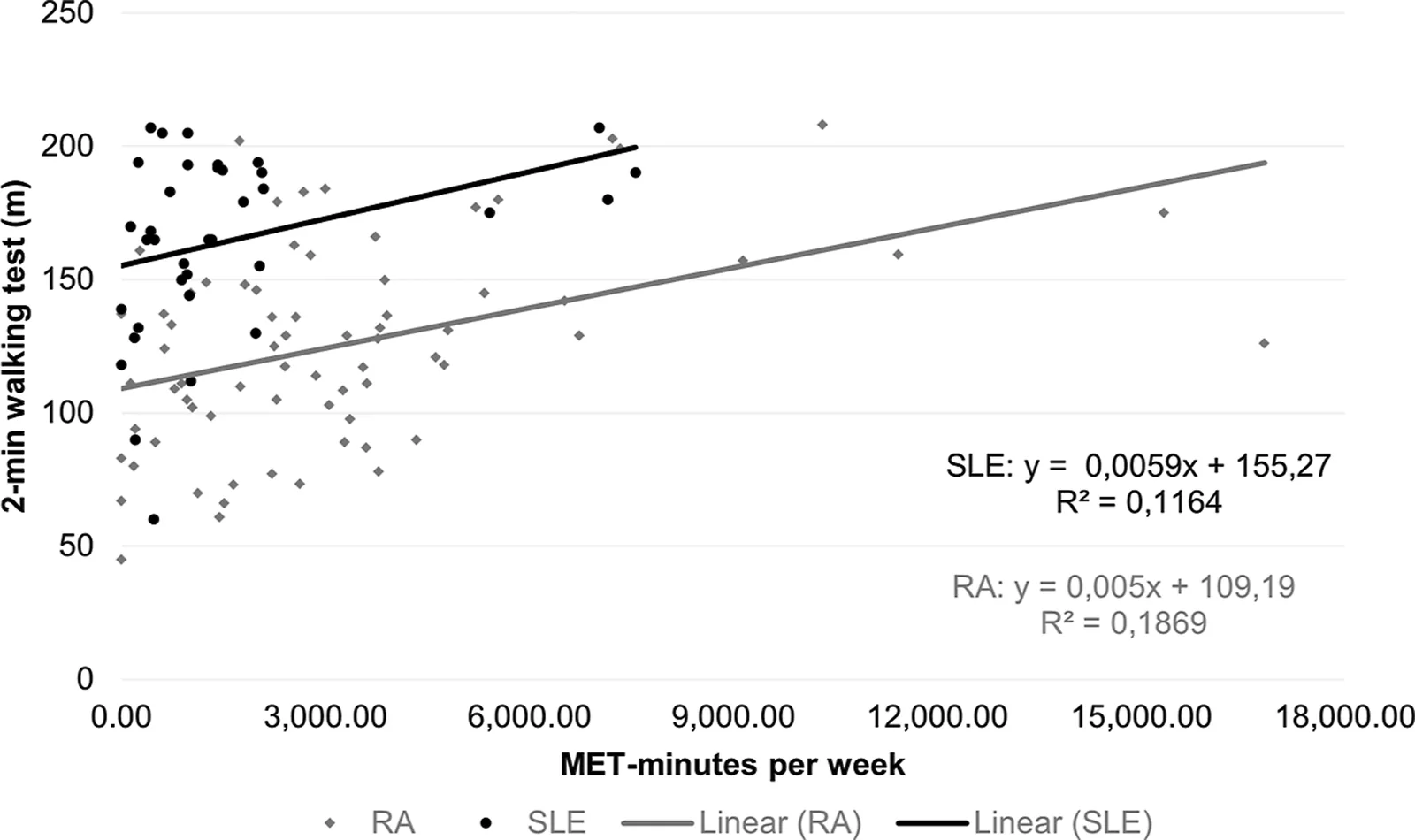
Association between weekly physical activity and 2-minute walk test performance in patients with rheumatoid arthritis and systemic lupus erythematosus. Scatter plots display individual data points, and solid lines represent linear regression fits for each disease group. RA, rheumatoid arthritis; SLE, systemic lupus erythematosus; MET, metabolic equivalent; 2MWT, 2-minute walk test.

**Table 3.** Psychological and clinical parameters in RA and SLE

Variable	Category	RA, %	SLE, %	χ^2^	*p* value
BDI-II (4 categories)	Minimal	79.7	73.4	1.813	0.612
​	Mild	10.2	11.7	​	​
​	Moderate	7.6	12.8	​	​
​	Severe	2.5	2.1	​	​
BDI-II (5 categories)	None	60.7	45.7	5.681	0.224
​	Minimal	18.8	27.7	​	​
​	Mild	9.4	12.8	​	​
​	Moderate	7.7	11.7	​	​
​	Severe	3.4	2.1	​	​
Fibromyalgia	Absent	85.0	72.3	4.659	0.031
​	Present	15.0	27.7	​	​
Smoking	No	82.5	90.4	3.156	0.076
​	Yes	17.5	9.6	​	​

Values are presented as percentages. *p* values are based on χ^2^ tests comparing both groups.

RA, rheumatoid arthritis; SLE, systemic lupus erythematosus; BDI-II, Beck Depression Inventory-II.

The standard IPAQ model in patients with RA demonstrated a good overall fit in the multivariable logistic regression analysis (Omnibus χ^2^ = 21.24, *p* = 0.002; Nagelkerke *R*^*2*^ = 0.63; classification accuracy: 93.2%). After adjustment, fibromyalgia was the only independent predictor of being physically inactive, being strongly associated with lower odds of being active (OR = 0.001, 95% CI < 0.001–0.289; *p* = 0.017; Table 4). Associations for SF-36 physical functioning, depressive symptoms, and disease activity did not remain statistically significant. Given the 59 complete RA cases, findings should be considered exploratory.

**Table 4.** Selected multivariable associations with physical activity in RA and SLE

Predictor	RA OR (95% CI)	*p* value	SLE OR (95% CI)	*p* value
Fibromyalgia	0.001 (<0.001–0.289)	0.017	0.374 (0.081–1.737)	0.209
SF-36 physical functioning	1.081 (0.993–1.176)	0.074	1.036 (0.991–1.082)	0.116
BDI-II	1.364 (0.956–1.945)	0.086	0.341 (0.110–1.061)	0.063
Disease activity (DAS28/SLEDAI)	0.417 (0.015–11.881)	0.101	–	–

Odds ratios (ORs) with 95% confidence intervals (CIs) are derived from multivariable logistic regression models. All estimates are adjusted for the full set of covariates included in each model. For RA, the standard IPAQ grouping and for SLE, the alternative IPAQ grouping was applied. Disease activity was not included in the final SLE model due to lack of significance. Complete model specifications are provided in Supplementary Table 5.

RA, rheumatoid arthritis; SLE, systemic lupus erythematosus; BDI-II, Beck Depression Inventory-II; SF-36, Short Form-36 Health Survey; IPAQ, International Physical Activity Questionnaire; DAS28, Disease Activity Score in 28 joints; SLEDAI, Systemic Lupus Erythematosus Disease Activity Index.

In SLE, no independent predictors were identified using the standard IPAQ grouping. In contrast, the alternative IPAQ model showed a significantly improved fit (Omnibus χ^2^ = 20.56, *p* = 0.015), with moderate explanatory power (Nagelkerke *R*^*2*^ = 0.32) and a classification accuracy of 76.3%. Within this model, fibromyalgia (OR = 0.374, *p* = 0.209) and depressive symptoms (OR = 0.341, *p* = 0.063) showed nonsignificant trends toward lower odds of being physically active, while SF-36 physical functioning was not independently associated with PA (Table 4). Model calibration was adequate for both final models, with nonsignificant Hosmer-Lemeshow tests (RA: *p* = 0.642; SLE: *p* = 0.716). Firth penalized logistic regression yielded comparable results, with fibromyalgia remaining significantly associated with physical inactivity (OR = 0.012, 95% CI: 0.00009–0.388, *p* = 0.010). Univariate regression results are provided in online supplementary Tables S3 and S4.

## Discussion

This study examined associations of PA in RA and SLE, while acknowledging that the cross-sectional design allows the identification of associations rather than causal relationships. Our findings demonstrate that associations with self-reported PA differ between the two diseases and depend strongly on the assessment of PA, including the applied IPAQ classification and complementary functional performance measures, a pattern that has been consistently reported in previous studies [29–31] and may provide insights into potential determinants to be examined in future research.

Despite more pronounced functional limitations and shorter walking distances in the 2-minute walk test (shown in Fig. 3), patients with RA reported higher levels of PA than those with SLE. This discrepancy between objectively assessed physical performance and subjectively reported activity highlights the complexity of PA behavior in inflammatory RDs and underlines the importance of considering subjective and objective assessment tools in daily clinical care. Disease-specific comorbidities, such as predominant joint involvement in RA, may limit comparability of 2-minute walk test performance between the diseases and affect the interpretation of between-group differences. The positive association observed in both disease groups between the subjective IPAQ and the 2-minute walk test performance supports the construct validity of self-reported activity measures. The explained variance suggests that multiple factors contribute to physical performance (shown in Fig. 3).

Lower PA levels in SLE, compared with RA, are consistent with higher levels of depressive symptoms observed in this population, as well as with previous reports, suggesting that systemic manifestations and a fluctuating disease course contribute to sustained physical inactivity [32, 33].

Regarding multivariable predictors of PA in RA, fibromyalgia was the only variable independently associated with physical inactivity in the fully adjusted multivariable model. This association persisted after accounting for functional impairment, depressive symptoms, disease activity, and sociodemographic factors and was consistent in a simplified RA model, supporting model stability. These findings are consistent with previous studies in which pain, disease activity, and functional limitations have been described as relevant barriers to PA in RA, although these factors did not retain independent significance in the fully adjusted model of the present study [34–36]. Notably, neither disease activity nor depressive symptoms retained an independent association with PA in the multivariable model. This association may reflect fibromyalgia-related symptom burden, potentially involving inflammatory and stress-related pathways, which could influence activity behavior [37]. It should also be considered that fibromyalgia involves mechanisms such as central sensitization, altered pain processing, and fatigue, which may contribute to the reduced PA [38, 39]. As these factors were not comprehensively assessed in this study, the underlying mechanisms could not be further explored. The extreme OR observed in the primary RA model likely reflects sparse data bias. Although the estimated effect was smaller in the Firth penalized logistic regression, fibromyalgia remained significantly associated with physical inactivity. Given the wide CIs, the magnitude of this association should be considered exploratory.

Although PA has been shown to reduce pain, improve function, and cardiovascular health [34, 40, 41], our findings suggest that general exercise recommendations may not sufficiently address disease-specific barriers to PA in RA patients with fibromyalgia and may therefore require individual adaptation. In patients with underlying RDs, fibromyalgia may overlap with other chronic pain conditions, and the observed association may reflect a broader pain-related symptom burden rather than a distinct diagnostic entity. In this context, pain may also be interpreted as chronic secondary pain rather than a separate comorbid condition. This conceptual overlap limits the specificity of fibromyalgia classification in this context.

In SLE, multivariable analysis including the alternative IPAQ grouping did not identify any independent predictors of PA. Although model fit was acceptable, none of the included variables retained statistical significance after full adjustment. Fibromyalgia and depressive symptoms showed weak, nonsignificant trends toward lower odds of being physically active, while functional status and fatigue were not independently associated with PA. Complete multivariable model specifications are provided in online supplementary Table 5. Previous studies have identified fatigue, pain, depressive symptoms, and impaired health-related quality of life as major barriers to PA in SLE [33, 42]. Our results extend this evidence by showing that these factors, although widely reported as relevant, may not consistently retain independent predictive value, thereby suggesting the need for individualized and multidimensional intervention strategies in SLE.

Overall, multivariable modeling revealed different association patterns between RA and SLE. These findings suggest that factors associated with PA in SLE are less distinct and more multifactorial than in RA, reflecting multisystemic disease manifestations [43–45]. Focusing on health-enhancing activity levels may allow a more sensitive identification of relevant differences in PA behavior in SLE, for which moderate activity alone does not necessarily translate into measurable benefits. Within the IPAQ framework, health-enhancing PA is primarily defined at the highest activity category, and existing studies suggest that this classification can serve as a useful orientation for patients with RDs [5, 27, 46].

Because estimates of PA are highly dependent on assessment and classification methods, examining the relationship between self-reported activity and objective functional performance is essential. Accordingly, the moderate association observed between self-reported PA and objective walking performance (shown in Fig. 3) in both disease groups reflects this approach and is in line with earlier findings, demonstrating limited agreement between questionnaire-based assessments and accelerometer-derived measures of PA [47–49]. In this context, PROMS provide complementary information that cannot be fully captured by clinical parameters alone, particularly in SLE, where the disease burden is multifactorial and difficult to quantify within routine clinical encounters [13, 50, 51]. Our findings therefore support the use of validated PROMs to provide individualized counseling and shared decision-making.

Several limitations should be considered when interpreting the results of this study. The cross-sectional, single-center design does not allow causal conclusions, increasing the risk of overinterpretation, particularly to potential causal relationships and the applicability of the findings to broader clinical settings. Differences in baseline characteristics such as age, sex distribution, functional status, and walking performance between RA and SLE cohorts may limit direct comparability and should be considered when interpreting between-group differences. PA was assessed using self-reported questionnaires, which may overestimate activity levels compared with objective measures and are subject to recall and reporting bias. This could contribute to overinterpretation. Objective physical performance was available only in a subset of patients, and fatigue was assessed only in the SLE cohort, limiting comparability and preventing evaluation of whether fatigue, which is potentially related to fibromyalgia, acts as a key determinant of reduced PA, particularly in RA [52]. As fibromyalgia was assessed as a dichotomous variable using the ACR 1990 criteria, the analysis captures the presence of fibromyalgia, but not differences in symptom burden or severity among affected patients. The smaller sample size in SLE may have reduced statistical power in multivariable analyses. In addition, residual confounding by unmeasured factors, such as medication use or socioeconomic characteristics, cannot be ruled out. Chronic disease damage, including structural joint damage and pulmonary involvement, was not assessed and may also have contributed to the observed association between fibromyalgia and PA. Another potentially relevant factor not assessed in this study is sarcopenia, which may influence PA and functional capacity and could limit the interpretation of the observed associations. In addition, the absence of complementary disease-specific indices and detailed organ involvement data may have limited the characterization of disease burden.

Strengths of this study include the parallel assessment of variables associated with PA in two distinct inflammatory RDs, the integration of PROMs with objective functional performance, and the use of multivariable models to account for potential confounding across clinical and psychological domains. Using an alternative IPAQ classification allowed a differentiated assessment of health-enhancing activity levels and confirmed comparable patterns and directions of associations.

In summary, our results suggest that associations with PA may differ between inflammatory RDs. In RA, fibromyalgia was associated with lower PA, which should be considered exploratory due to the small sample of 59 cases. This association warrants further investigation of pain-related comorbidities to support sustained engagement in PA. Further research in larger cohorts is required to determine their relevance for targeted interventions in clinical practice. In SLE, PA behavior appears to be shaped by a more complex interplay of functional and psychological factors, which becomes particularly evident when health-enhancing activity levels are considered. Further research is warranted to determine how these disease-specific patterns can be translated into individualized strategies for promoting PA in inflammatory RDs.

These findings support current EULAR recommendations advocating individualized, patient-centered PA interventions that account for comorbidities, functional capacity, and personal preferences [5, 45, 52, 53]. While targeted strategies addressing fibromyalgia may be particularly relevant for patients with RA, promoting PA in SLE likely requires broader, multidimensional approaches rather than a focus on single disease-related factors. At the same time, PA promotion should remain a priority, as even modest levels of activity contribute to reducing inactivity, particularly when guideline targets cannot be fully achieved [54, 55]. In this context, PROMs may complement guideline-based recommendations by supporting more individualized PA counseling, particularly in patients with relevant comorbidities.

## Acknowledgments

The authors thank Dr. Holger Bogatsch, Institute for Medical Informatics, Statistics, and Epidemiology (IMISE), Leipzig University, for statistical advice. During the preparation of this manuscript, the authors used generative artificial intelligence for language editing technical support with statistical coding. The authors reviewed and edited the content and take full responsibility for the manuscript.

## Statement of Ethics

Both studies were approved by the Local Ethics Committee of Leipzig University Medical Faculty (Approval: RA 363/18-ek; SLE 035/16-ek). Written informed consent was obtained from all participants prior to study inclusion.

## Conflict of Interest Statement

The authors have no conflicts of interest to declare.

## Funding Sources

This study was not supported by any sponsor or funder.

## Author Contributions

Justus Mörstedt contributed to data preparation, analysis, interpretation, and manuscript drafting. Benjamin Graul and Lev Lipovsky contributed to data acquisition. Olga Seifert contributed to conceptualization, data interpretation, and manuscript preparation. All authors read the final version of the manuscript and agreed to its publication.

## Data Availability

The data that support the findings of this study are not publicly available due to their containing information that could compromise the privacy of research participants but are available from the corresponding author (justusmoerstedt@gmail.com) upon reasonable request.
